# Exploring Gender Disparities in the Prevalence and Clinical Characteristics of Atherosclerotic Cranial Stenosis

**DOI:** 10.7759/cureus.45809

**Published:** 2023-09-23

**Authors:** Saeed A Alqahtani

**Affiliations:** 1 Neurology, College of Medicine, King Khalid University, Abha, SAU

**Keywords:** extracranial stenosis, intracranial stenosis, smoking habits, obesity, metabolic syndrome, dyslipidemia, gender disparities, ischemic stroke, atherosclerotic cranial stenosis (acs)

## Abstract

Background

Atherosclerotic cranial stenosis (ACS) is a significant contributor to vascular events, including ischemic strokes. While early clinical studies suggested a divergence in the distribution of intracranial and extracranial stenosis between genders, recent evidence has highlighted the complexity of these disparities. Therefore, this study aims to investigate gender differences in the prevalence and clinical characteristics of atherosclerotic cranial stenosis in patients admitted with stroke.

Methods

This cross-sectional study was conducted at a tertiary care hospital located in the Southern Region of Saudi Arabia between June 2022 and December 2022. It included patients of all age groups who had been diagnosed with an ischemic stroke during the study period. Data were collected from electronic health records and medical archives, and data analysis was performed using Statistical Package for the Social Sciences (SPSS version 26, IBM Inc., Chicago, IL, USA).

Results

In our study, 201 stroke patients were analyzed, with 161 (80.09%) identified as having atherosclerotic stenosis. Of these, 57.8% were male, and 42.2% were female. Gender disparities were evident, with higher stenosis prevalence in males (46.27% vs. 33.83% in females). Significant gender differences were observed in dyslipidemia (p = 0.013), metabolic syndrome (p = 0.019), and smoking habits (p < 0.001). Males exhibited higher rates of extracranial stenosis (p = 0.012) and combined stenosis (p = 0.009) compared to females; however, females exhibited higher rates of intracranial stenosis (p = 0.013). Further analyses revealed significant associations in dyslipidemia (adjusted odd ratio (AOR): 0.245, p = 0.004), metabolic syndrome (AOR: 5.159, p = 0.006), obesity (AOR: 8.085, p = 0.016), smoking habits (AOR: 0.002, p < 0.001), and intracranial stenosis (AOR: 5.667, p = 0.005) within the female cohort. Conversely, age, hypertension, diabetes mellitus, ischemic heart disease, and extracranial stenosis did not show statistically significant associations in females (p > 0.05).

Conclusion

We observed a substantial presence of atherosclerotic cranial stenosis, with males showing higher rates, and identified significant gender-related variations in dyslipidemia, metabolic syndrome, and smoking habits as important factors. This highlights the necessity of tailoring ACS assessment and treatment by considering gender-specific risk factors and clinical characteristics for improved patient care and stroke management.

## Introduction

Atherosclerotic cranial stenosis (ACS) constitutes a significant risk factor for vascular incidents, with its involvement established in approximately 8% to 10% of all cases of ischemic strokes. Additionally, ACS emerges as a significant contributor to recurrent strokes and vascular mortality [1]. It is important to note that the prevalence of intracranial stenosis does not exhibit a uniform distribution across demographic groups. Research has indicated that this condition appears to be more prevalent among individuals of African, Hispanic, and Asian descent compared to those of Caucasian ethnicity [1].

Stroke incidence exhibits a 33% higher occurrence among males, and on average, males experience their first stroke approximately 4.3 years earlier than females [2]. Early clinical studies initially reported a female predominance of intracranial stenosis and a male predominance of extracranial stenosis [3]. However, more recent clinical evidence has revealed a male predominance of intracranial stenosis [4]. The prevalence of cerebrovascular atherosclerosis also differs between males and females, although the findings remain inconsistent. An autopsy study demonstrated that from the fourth to the sixth decade, males display a higher frequency and severity of atherosclerosis than females. However, in populations aged 65 years and above, the frequency of atherosclerosis aligns between the two genders [5]. Furthermore, a study conducted in China observed that males are more susceptible to middle cerebral artery stenosis than females [6], while the International Atherosclerosis Project noted that carotid and vertebral arteries are more commonly affected in males than in females [7]. The prevalence of ACS, along with its associated risk factors and clinical presentations, may significantly vary between men and women, attributable to hormonal, genetic, and sociocultural factors. These disparities could have far-reaching consequences for early detection, diagnosis, and treatment and ultimately influence patient outcomes and healthcare resource allocation.

The study of gender disparities in healthcare has become increasingly pertinent as we strive for equitable healthcare outcomes. Despite this knowledge, there is a paucity of research that specifically examines how gender influences the occurrence and clinical features of ACS. This research endeavors to address this critical knowledge gap by conducting a systematic exploration of gender disparities in the prevalence and clinical characteristics of ACS. By conducting this research, we seek to uncover vital insights that can inform healthcare providers, policymakers, and researchers about the gender-specific dimensions of ACS. Understanding these nuances will enable the development of more effective prevention strategies, diagnostic tools, and treatment approaches, ultimately leading to improved patient care and healthcare equity in the context of cerebrovascular diseases.

## Materials and methods

This cross-sectional study was conducted between June 2022 and December 2022 at a tertiary care hospital located in the southern region of Saudi Arabia. Ethical approval for the study was obtained from the Institutional Review Board Committee of the Ministry of Health Affairs-Aseer Region.

The study included patients of all ages diagnosed with ischemic strokes during the study period. Patients with incomplete medical records or patients who had experienced a hemorrhagic stroke or a transient ischemic attack (TIA) were intentionally excluded from the subsequent analysis.

Data were collected from electronic health records, hospital databases, and medical archives. The following information was collected: demographic information (age, gender, and race/ethnicity). Risk factors for ischemic stroke (hypertension, diabetes, hyperlipidemia, smoking, and ischemic heart disease). Clinical characteristics of ischemic stroke (location, severity, and complications).

The diagnosis of ischemic stroke was confirmed through a comprehensive evaluation of clinical symptoms and corroborated by neuroimaging techniques such as computed tomography (CT) or magnetic resonance imaging (MRI), all within the predetermined timeframe designated for the study.

Data analysis was performed using the Statistical Package for the Social Sciences (SPSS version 26, IBM Inc., Chicago, IL, USA). Descriptive statistics, including frequencies, percentages, means, and standard deviations, were calculated to summarize the data. The chi-squared test was employed to evaluate associations between categorical variables. Multivariate logistic regression analysis was employed to control potential confounding variables and assess the independent association between gender and demographic and clinical characteristics. A P-value <0.05 was considered statistically significant.

## Results

In our study, a total of 201 patients diagnosed with stroke were included for analysis, of which 161(80.09%) were identified as having atherosclerotic stenosis. Out of 161, 93(57.8%) were male, while 68(42.2%) were female. Age distribution shows that 106(65.8%) of participants were aged over 55 years, with the 46-55 years age group representing 28(17.4%) of the sample. Most of the participants had hypertension 130(80.7%), diabetes mellitus 80(49.7%), dyslipidemia 84(52.2%), metabolic syndrome 62(38.5%), and a history of ischemic heart disease 50(31.1%). Additionally, obesity was observed in 39(24.2%) of participants, and smoking was reported by 26(16.1%). Table 1 provides a comprehensive overview of the participant demographics.

**Table 1:** Demographic characteristics of the study population (n = 161).

Variable	Frequency	Percentages
Gender		
Male	93	57.8
Female	68	42.2
Age group		
<25 years	12	7.5
26–35 years	03	1.9
36–45 years	12	7.5
46–55 years	28	17.4
>55 years	106	65.8
Risk factors		
Hypertension	130	80.7
Diabetes mellitus	80	49.7
Dyslipidemia	84	52.2
Metabolic syndrome	62	38.5
Ischemic heart disease	50	31.1
Obesity	39	24.2
Smoking	26	16.1

In terms of stenosis types, the majority of cases 92(57.1%) exhibited intracranial stenosis, while 39(24.2%) were categorized as extracranial stenosis. Interestingly, 30(18.6%) of cases displayed both intracranial and extracranial stenosis simultaneously. Regarding the degree of stenosis, the findings revealed a diverse range of severity levels. Nearly half of the cases 73(45.3%) fell into the 50-69% stenosis category, while 41(25.5%) exhibited stenosis in the 70-89% range. Severe stenosis (90-99%) was observed in 22(13.7%) of cases, and 25(15.5%) of the population had complete occlusion (100% stenosis) (Table 2).

**Table 2:** Arteriosclerotic stenosis and severity distribution in the study population.

Variable	Frequency	Percentages
Stenosis		
Intracranial stenosis	92	57.1
Extracranial stenosis	39	24.2
Both	30	18.6
Degree of stenosis		
50-69%	73	45.3
70-89%	41	25.5
90-99%	22	13.7
100%	25	15.5

**Figure 1:**
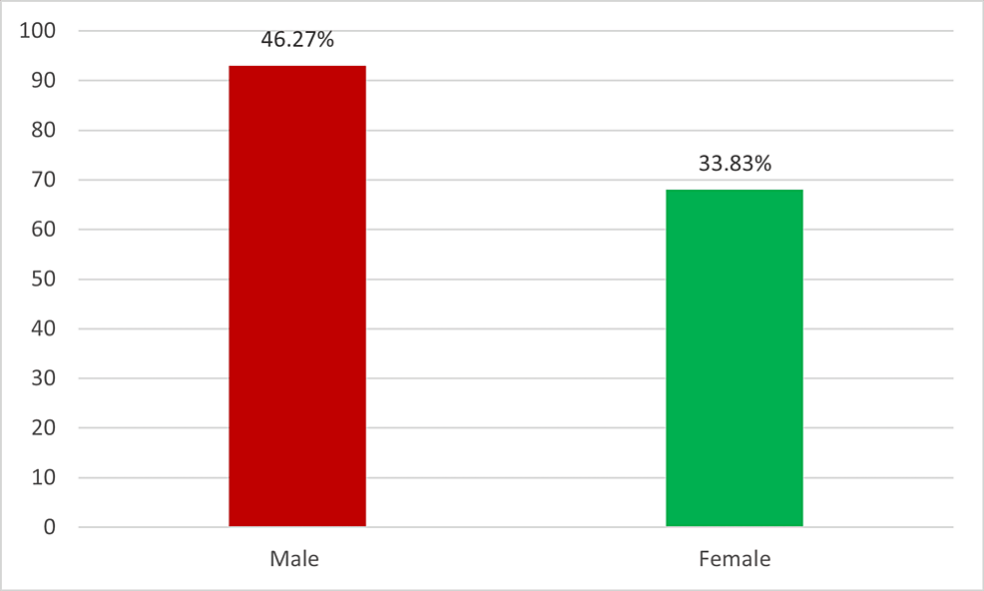
Prevalence of atherosclerosis stenosis in patients with stroke.

Among the study participants, the prevalence of stenosis in males was notably higher, standing at 46.27%, whereas in females, it was observed to be 33.83% (Figure 1).

Table 3 displays the gender-based distribution of demographic and clinical characteristics. Statistically significant gender differences were observed in the prevalence of dyslipidemia (p = 0.013), with 56(60.2%) of males and 28(41.2%) of females affected, as well as in the occurrence of metabolic syndrome (p = 0.019), with 29(31.2%) in males and 33(48.5%) in females. Moreover, significant gender disparities were evident in smoking habits (p < 0.001), with 25(26.9%) of males and only 01(1.5%) of females reporting smoking. Additionally, differences were noted in the prevalence of stenosis: males have higher rates of intracranial stenosis compared to extracranial stenosis, while females have higher intracranial stenosis than males. Males exhibited higher rates of intracranial stenosis 44(47.3%) vs. 48(70.6%), p = 0.013, extracranial stenosis 28(30.1%) vs. 11(16.2%), p = 0.012, and combined stenosis 21(22.6%) vs. 09(13.2%), p = 0.009 compared to females.

**Table 3:** Gender-based distribution of demographic and clinical characteristics of study participants.

Variables	Gender		P-value
	Male (93)	Female (68)	
Age (mean±SD)	62.17±17.39	61.29±21.08	0.773
Hypertension	72(77.4)	58(85.3)	0.147
Diabetes mellitus	44(47.3)	36(52.9)	0.293
Dyslipidemia	56(60.2)	28(41.2)	0.013
Metabolic syndrome	29(31.2)	33(48.5)	0.019
Ischemic heart disease	25(26.9)	25(36.8)	0.122
Obesity	17(18.3)	22(32.4)	0.31
Smoking	25(26.9)	01(1.5)	<0.001
Stenosis			
Intracranial	44(47.3)	48(70.6)	0.013
Extracranial	28(30.1)	11(16.2)	0.012
Both	21(22.6)	9(13.2)	0.009

Table 4 showing multivariant logistic regression, dyslipidemia demonstrated a significant association, with an AOR of 0.245 (95% CI: 0.094, 0.638, p = 0.004). Conversely, metabolic syndrome showed a significant positive association, with an AOR of 5.159 (95% CI: 1.613, 16.497, p = 0.006). Additionally, obesity exhibited a significant positive association, with an AOR of 8.085 (95% CI: 1.484, 44.035, p = 0.016). Smoking habits displayed a highly significant association, with an AOR of 0.002 (95% CI: 0.00, 0.032, p < 0.001). Intracranial stenosis also revealed a significant positive association, with an AOR of 5.667 (95% CI: 1.668, 19.256, p = 0.005). Conversely, age, hypertension, diabetes mellitus, ischemic heart disease, and extracranial stenosis did not exhibit statistically significant associations with the outcome in the female cohort (p > 0.05).

**Table 4:** Multivariate logistic regression analysis of factors associated with atherosclerotic cranial stenosis.

Female	AOR (95% CI)	P-value
Age	1.006 (0.980, 1.033)	0.661
Hypertension	0.753 (0.219, 2.587)	0.653
Diabetes mellitus	2.374 (0.951, 5.926)	0.064
Dyslipidemia	0.245 (0.094, 0.638)	0.004
Metabolic syndrome	5.159 (1.613, 16.497)	0.006
Ischemic heart disease	1.835 (0.626, 5.375)	0.269
Obesity	8.085 (1.484, 44.035)	0.016
Smoking	0.002 (0.00, 0.032)	0.000
Intracranial stenosis	5.667 (1.668, 19.256)	0.005
Extracranial stenosis	0.623 (0.158, 2.445)	0.497

## Discussion

Our study delved into the prevalence and clinical characteristics of atherosclerotic cranial stenosis (ACS) among stroke patients, shedding light on gender disparities in this condition. Males exhibited a notably higher prevalence of ACS compared to females, with 57.8% of ACS cases being male. This observation aligns with broader stroke epidemiology trends, where males tend to experience strokes at a higher rate and often at a younger age than females. Males exhibited a notably higher prevalence of extracranial circulation atherosclerosis (ECAS) in comparison to females, whereas females demonstrated a significantly higher proportion of intracranial circulation atherosclerosis (ICAS) cases relative to males (47.3% vs. 70.6%, p = 0.013). Previous research has established that atherosclerosis affecting extracranial circulation is more frequently observed in males than females. This discrepancy is often attributed to the absence of the protective effects of estrogen in males and increased exposure to vascular risk factors [8]. This observation is consistent with the findings of a meta-analysis conducted on an Asian population, which emphasized a stronger association between the female sex and ICAS as opposed to ECAS [9]. Furthermore, an investigation conducted within a Chinese community population, focusing on asymptomatic ICAS cases, disclosed that males exhibited a lower prevalence of ICAS, while females exhibited a higher likelihood of ICAS occurrence compared to males [10]. 

However, it is worth noting that conflicting results have been reported in prior studies. A population-based multicenter study, for instance, indicated that ICAS prevalence was higher in males (38.5%) than in females (31.7%) [11].

Our study revealed a compelling association between ACS and dyslipidemia among male stroke patients. This finding is supported by various studies [12,13]. In a comprehensive meta-analysis involving 64 randomized controlled trials aimed at examining the association between initial serum triglyceride (TG) levels and cerebrovascular incidents, it was ascertained that for every 10 mg/dL increment in baseline serum TG levels, there was an estimated 5% elevation in the risk of experiencing all types of strokes [14].

The prevalence of smoking was higher among males compared to females, while metabolic syndrome exhibited a higher prevalence in the female cohort. It is noteworthy that smoking has been consistently identified as a significant risk factor associated with carotid atherosclerosis, potentially hastening the onset or progression of atherosclerotic conditions [15]. Notably, smoking constitutes a well-established and potent risk factor for the development of carotid atherosclerosis and ischemic stroke.

Furthermore, it is essential to consider the impact of metabolic syndrome on cerebral arterial atherosclerosis stenosis, particularly in patients with a history of stroke. However, the influence of metabolic syndrome on individuals without a history of stroke remains a subject of debate and ambiguity. Prior investigations conducted within hospital-based settings have reported metabolic syndrome as an independent risk factor for stroke [16-18].

Metabolic syndrome holds significant relevance due to its gender-specific prevalence patterns. Generally, females exhibit a higher prevalence of metabolic syndrome compared to males, while conversely, smoking is more prevalent among males compared to females. This gender-based contrast underscores the importance of understanding the interplay between metabolic syndrome and smoking, as it highlights distinct health challenges and risk factors faced by each gender. Recognizing these disparities is crucial for tailored public health interventions and strategies aimed at addressing complex health outcomes.

The limitations of this study include its single-center design, which may limit the generalizability of findings beyond the specific geographic region. Additionally, the retrospective nature of data collection raises the possibility of incomplete or missing data, potentially impacting the accuracy of the analysis.

## Conclusions

This study concludes that there is a disparity in atherosclerotic cranial stenosis (ACS) rates between males and females, with males exhibiting a higher incidence. Our study unearthed substantial gender-related disparities in key risk factors, particularly in the prevalence of dyslipidemia, metabolic syndrome, and smoking habits. These factors are not only crucial determinants of ACS development but also have significant implications for stroke risk and management. Recognizing these gender-related variations underscores the imperative need to tailor ACS assessment and therapeutic strategies, accounting for these distinctive risk profiles and clinical characteristics.
